# Functional Characterization of *MaZIP4*, a Gene Regulating Copper Stress Tolerance in Mulberry (*Morus atropurpurea* R.)

**DOI:** 10.3390/life12091311

**Published:** 2022-08-26

**Authors:** Yisu Shi, Qiaonan Zhang, Lei Wang, Qiuxia Du, Michael Ackah, Peng Guo, Danyan Zheng, Mengmeng Wu, Weiguo Zhao

**Affiliations:** Key Laboratory of Silkworm and Mulberry Genetic Improvement, Ministry of Agriculture, School of Biology and Technology, Jiangsu University of Science and Technology, Zhenjiang 212018, China

**Keywords:** phylogenetic, virus-induced gene silencing, transgenic lines, physiological and biochemical analysis

## Abstract

*ZIP4* (zinc transporter 4) plays important roles in transporting Cu^2+^ ions in plants, which may contribute to the maintenance of plant metal homeostasis in growth, plant development and normal physiological metabolism. However, *ZIP4* transporters have not been described in mulberry and the exact function of *ZIP4* transporters in regulating the homeostasis of Cu in mulberry remains unclear. In this study, a new *ZIP4* gene (*MaZIP4*) was isolated and cloned from *Morus atropurpurea* R. Phylogenetic analysis of amino sequences suggested that the amino-acid sequence of the *MaZIP4* protein shows high homology with other *ZIP4* proteins of *Morus notabilis*, *Trema orientale*, *Ziziphus jujube* and *Cannabis sativa*. In addition, a *MaZIP4* silenced line was successfully constructed using virus-induced gene silencing (VIGS). The analysis of *MaZIP4* expression by quantitative real-time PCR in mulberry showed that the level of *MaZIP4* expression increased with increasing Cu concentration until the Cu concentration reached 800 ppm. Relative to the blank (WT) and the negative controls, malondialdehyde (MDA) levels increased significantly and rose with increasing Cu concentration in the *MaZIP4* silenced line, whereas the soluble protein and proline content, superoxide dismutase (SOD) and peroxidase (POD) activities of these transgenic plants were lower. These results indicated that *MaZIP4* may play an important role in the resistance of mulberry to Cu stress.

## 1. Introduction

Although copper (Cu) is an essential nutrient for plant growth and development, above certain physiological levels it can be toxic. It participates in numerous physiological processes and is an essential cofactor for many metalloproteins [1]. For example, Cu activates many enzymes in plants that are involved in lignin synthesis, and it is the key to the formation of chlorophyll for photosynthesis [2,3,4]. In addition, it is essential in plant respiration and assists in the plant metabolism of carbohydrates and proteins [2]. Another important function of Cu is to promote the development of flower organs and to intensify flower coloring [5]. Cu is one of the micronutrients needed by plants and the ideal range for Cu in the tissue is 20 times lower than that of iron [6]. Excess Cu may have a negative impact on plant growth and quality [4], and impairs leaf Cu concentration, gas exchange and protein profiles [7]. Excess Cu in the growth medium can restrict tap root growth by burning the root tips and thereby promoting lateral root growth [8]. High concentrations of Cu can compete with plant uptake of Ca, Mg, K, Zn and Fe, initially resulting in greener new growth than normal, but later to the exhibition of iron or other micronutrient deficiencies [8]. The continued exposure to excess Cu toxicity can reduce aerial branching and lead to further reductions in plant health. Plants must therefore have evolved appropriate strategies to maintain Cu homeostasis in response to different environmental Cu levels. Such strategies must prevent the accumulation of the metal in its reactive form within detoxification pathways and ensure that the element is properly transported for storage or for the biosynthesis of target metalloproteins. Although there has been substantial recent research on the mechanisms involved in Cu acquisition and transport into and within cells in yeast and other eukaryotic organisms, including *Arabidopsis thaliana* [9], these processes in plants remain incompletely understood in plant systems. Nevertheless, several families of heavy metal transporters involved in the maintenance of intracellular heavy metal homeostasis in plants have been identified [2,10].

ZIP transporters are divalent metal transporters, which have been identified in a variety of plant species, especially dicots such as *Arabidopsis* and soybean [10]. They are responsible for transporting various metal cations into the cytoplasm, such as Zn^2+^, Mn^2+^, Fe^2+^/Fe^3+^, Cd^2+^, Co^2+^, Ni^2+^ and Cu^2+^. It has been reported that ZIP proteins are involved in cellular uptake of Zn^2+^. *ZIP* transporters contain eight transmembrane domains and a histidine-rich variable loop between TM3 and TM4 [11]. There are 14 additional members of the *ZIP* family in *Arabidopsis* [12]. It has been shown that *AtZIP2* and *AtZIP4* can supplement the growth defects of Cu and Zn transport mutants in yeast [13,14]. Expression of the two genes is upregulated in *Arabidopsis* in deficiency of Cu and Zn, but not Fe [15]. In addition, *AtZIP2* has been proposed to participate in Cu acquisition by *Arabidopsis* roots. In the model legume *Medicago truncatula* L., six cDNA encoding *ZIP* family members have been identified, and their ability to complement yeast metal-absorption mutants have been tested [16]. Furthermore, according to the differential expression analysis of mRNA in response to different transition metal concentrations, a role for ZIP proteins in the maintenance of metal homeostasis was proposed [17]. In summary, the differential preferences of ZIP family members for divalent metals suggest that ZIP2 and ZIP4 proteins may play important roles in transporting Cu^2+^ ions. However, their role of these proteins in Cu transport needs to be confirmed and further investigated. 

Mulberry (*Morus. atropurpurea* R.) is an economically important perennial tree in China that is widely distributed in the northern temperate regions. As the sole food source of the domesticated silkworm, mulberry cultivation is crucial to the development of sericulture, and has many other important economic and ecological values [18]. Mulberry produces delicious fruits with medicinal value in the treatment of hypertension, oral and dental diseases, diabetes, arthritis and anemia. In addition, the fruit are also used in production of jams, juices, liquors, natural dyes and in the cosmetics industry. Relative to other plant species, mulberry shows high tolerance to multiple abiotic environmental factors in China, such as low temperature, high salinity, waterlogging and high soil concentrations of heavy metal ions [19,20,21]. Despite this tolerance, an excess of Cu in mulberry has been shown to disturb the cellular redox environment in young leaves, accelerate the rate of leaf senescence and damage roots. In addition, a deficiency in Cu is harmful to mulberry through the aggravation of oxidative stress through an enhanced generation of reactive oxygen species (ROS) and disturbed redox coupling [22]. Several families of heavy metal transporters are thought to be involved in the maintenance of intracellular Cu homeostasis in plants, including ZIP, COPT, P_IB_-ATPase, ATX, CCS and YSL [2]. ZIP family proteins are thought responsible for the uptake and allocation of many micronutrients including Fe, Mn, Zn and Cu [10,15]. OsZIP1, as a metal efflux transporter, limits the accumulation of excessive Zn, Cu and Cd in rice [23]. AtZIP2 has been implicated in Cu homeostasis and AtZIP4 was demonstrated to be involved in Cu transport in *Arabidopsis* [14]. However, the molecular roles of these heavy metal transporters have not been described in mulberry. Information on the role of the Cu transporters in mulberry and how the expression can be regulated by Cu concentrations may help to cultivate Cu-resistant plants, this expanding the practical planting range of mulberry.

Virus-induced gene silencing (VIGS) is a technique based on RNA interference to construct gene silencing lines, which is widely used to explore gene function [24,25]. Compared with other transgenic techniques, VIGS offers unique advantages in terms of a short cycle time, ease of operation, no need to build stably transformed plants and low costs. In this study, *MaZIP4* was associated with the response to Cu stress in mulberry through comparative transcriptome analysis. *MaZIP4* gene silencing by VIGS technology was subsequently performed to analyze the function of this gene.

## 2. Materials and Methods

### 2.1. Sample Collection, RNA Extraction and Sequencing

**Figure 1:**
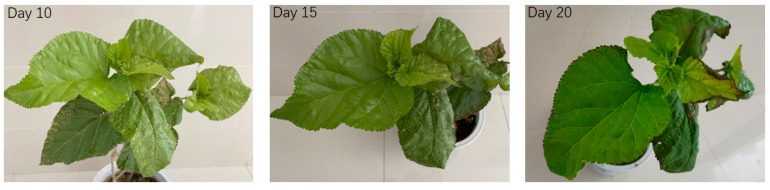
Mulberry leaves collected after 10, 15 and 20 days of stress response at 200 ppm CuSO_4_.

The mulberry (*M. atropurpurea* R.) materials used in this research were obtained from the sericulture research institute of Chinese Academy of Agricultural Sciences. The seedlings were transplanted in pots containing vermiculite and loamy soil (pH 6.5). seedlings with a length of 20 cm and consistent growth status were selected, three of which were treated with 500 mL of MS culture solution containing either 0 (control), or 200 ppm CuSO_4_ and grown for a further 20 days. All selected plants were watered every day. Three seedlings were selected from each of the experimental and control groups, representing three biological replicates. Leaf samples were collected from plants 1, 3, 5, 10, 15 and 20 days after Cu treatments. After 20 days of stress incubation, mulberry leaves showed yellowing and shrinkage, and burn marks appear on the leaf edges (Figure 1). For the expression analysis of *MaZIP4*, seedlings were selected as above and three biological replicates were treated with 0, 100, 200, 400 or 800 ppm CuSO_4_, following by daily watering for a further five days, after which young leaves were collected from each treatment group. In all cases, the collected leaves were immediately frozen in liquid nitrogen and stored at −80 °C for later RNA extraction. 

The total RNA of leaf samples was extracted using a reagent RNAiso Plus (Takara, Shanghai, China). The quality and quantity of total RNA were determined using an Agilent 2100 Bioanalyzer (Agilent Technologies, Palo Alto, CA, USA) and a NanoDrop 1000 spectrophotometer, respectively. 

RNA sequence libraries of Cu-treated and untreated leaf samples were prepared at the Novogene biotechnology company in Beijing, China. 1 μg total RNA from each sample was used for the construction of a RNA sequence library using NEBNext^®^UltraTM RNA Library Prep Kit from Illumina^®^ (NEB, Newburyport Tpke, Rowley, MA, USA) following the manufacturer’s specifications and index codes were added to attribute sequences to each sample.

### 2.2. Transcript Quantification and Differential Expression Analysis

Raw RNA-Seq reads were processed through in-house Perl scripts for trimming adapters, reads containing poly-N as well as low-quality bases from the reads ends. The clean paired-end reads of high quality were aligned to the reference genome for mulberry (https://morus.swu.edu.cn/morusdb/datasets, accessed on 10 November 2017) using Hisat2 v2.0.5 [26]. The mapped reads of each sample were assembled by StringTie (v1.3.3b) (https://github.com/gpertea/stringtie, accessed on 3 August 2022). The FPKM of each gene was calculated based on the read counts mapped to the gene calculated by featureCounts v1.5.0-p3 and the length of the gene. The DESeq2 R package (1.16.1) was used to perform the differential expression analysis between the Cu-treatments and the control groups (three biological replicates per group). *p*-Values were adjusted using the Benjamini and Hochberg’s approach [27] for controlling the false discovery rate in multiple testing and genes with an adjusted *p*-value < 0.05 and an absolute fold change of ≥2 were considered differentially expressed. In order to validate the result, differential expression analysis was performed using the edgeR R package (3.18.1). 

### 2.3. Cloning and Sequence Analysis of the ZIP4 Gene Homolog in Mulberry (MaZIP4)

9 μg of total RNA was used as template for reverse transcription with M-MLV reverse transcriptase (Takara Bio, Beijing, China). The cDNA was used as template for amplification of *MaZIP4* gene using the PCR primers, *MaZIP4*-F: 5’-ATGGCGAATACAAGTTGCCAGAGC-3’ and *MaZIP4*-R: 5’-TCAAGCCCAAATAGCTAATGAAGAC-3’, which were designed by Oligo7 based on the coding sequence of *MaZIP4* gene obtained from transcriptome data prepared above. The purified DNA fragment amplified by PCR was ligated into the pMDTM18-T Vector and amplified by transformation of *E. coli* TOP 10 cells (Takara Bio, Beijing, China). The bacterial solution was sequenced with an automated DNA sequencer (Sangon Biotech, Shanghai, China). DNASTAR was used to integrate the sequence fragments of upstream and downstream to obtain the whole sequence of the cloned *MaZIP4*.

DNAMAN was used to perform the sequences analysis of *MaZIP4*. The online software ExPASy (http://web.expasy.org/protparam/, accessed on 1 July 2020) was used to predict theoretical isoelectric point (pI) and the molecular weight of protein encoded by *MaZIP4*. The molecular modeling of protein encoded by *MaZIP4* was predicted using SWISS-MODEL (http://swissmodel.expasy.org/, accessed on 1 June 2022). Homologous sequences were searched from the NCBI database using BLAST [28]. Alignments of DNA/protein sequences were carried out using BLAST/Protein BLAST and non-rooted phylogenetic tree drawings were carried out using MEGA6.0. 

### 2.4. Expression Analysis of MaZIP4 under Different Degree of Cu Stress

The qRT-PCR method was used to analyze the different expression levels of *MaZIP4* in response to Cu stress [29]. cDNA synthesis used 9 μg of total RNA and M-MLV Reverse Transcriptase (TaKaRa-Bio, China) with oligo (dT)18 primer. The β-actin gene [30] was amplified using the primers: β-actin-F; 5′-AGCAACTGGGATGACATGGAGA-3′ and β-actin-R; 5′-CGACCACTGGCGTAAAGGGA-3′ as the internal reference gene. qRT-PCR was performed on a ABI Quant Studio 6 Flex (Maywood Ave, CA, USA) instrument with gene-specific forward and reverse primers (q*MaZIP*-F:5′-TGCTGCATTATCCTTCCACCA-3′, q*MaZIP*-R: 5′-AAGCAATGGCAGTCCCAA-3′) designed by Oligo7 based on the sequence of the cloned *MaZIP4* gene. 4 µL cDNA was used as the template of qRT-PCR. SYBR Green RT-PCR was performed according to FastStart universal SYBR Green Master Mix Kit (Novoprotein, Nanjing, China) specification in the LightCycler ^®^96 real-time PCR system (Novoprotein, Nanjing, China). The qRT-PCR conditions were 95 °C for 1 min followed by 35 cycles of 95 °C for 20 s, 57 °C for 20 s and 72 °C for 30 s. PCR specificity was checked by melting curve analysis, and data were calculated using the 2^−ΔΔCt^ method [31]. Standard errors and standard deviations were also calculated simultaneously.

### 2.5. Functional Analysis of MaZIP4 in Mulberry

#### 2.5.1. Transient Transformation of Mulberry Leaves for *MaZIP4* Repression

According to the obtained sequence of *MaZIP4*, the primers *MaZIP4*-F1 (5′-GGGGTACCATGGCGAATACAAGTTGCCAGAGC-3′) and *MaZIP4*-R1 (5′-GCTCTAGATCAAGCCCAAATAGCTAATGAAGAC), each including a Kpn I or Xbal I restriction enzyme site at their 5′ end, respectively.) were used for the PCR amplification of *MaZIP4*. The PCR products were then ligated into the pMDTM18-T Vector, which was used to transform E. coli TOP 10 cells. The recombinant plasmid was then isolated and the *MaZIP4* insert isolated after digestion with Kpn I and Xba I and inserted into the similarly digested pTRV2 vector with T4 DNA ligase to construct the pTRV2-*MaZIP4* vector. The pTRV2-*MaZIP4* vector was then transferred into *Agrobacterium tumefaciens* GV3101 using the freeze-thaw method [32]. The primers *MaZIP4*-F1 and *MaZIP4*-R1 were used to PCR amplify the *MaZIP4* insert in the isolated pTRV2-*MaZIP4* vector for sequence verification. pTRV1 and pTRV2 were used as negative controls.

*A. tumefaciens* transformed with pTRV1, pTRV2 or pTRV2-*MaZIP4* was resuspended in transient transformation buffer (150 mM AS (acetosyringone), 10 mM MES, 10 mM MgCl_2_). Cultures transformed with pTRV1 and pTRV2-*MaZIP4* were mixed in equal volumes for gene silencing. A similar mixture of pTRV1 and pTRV2 was prepared as a mock control (Young et al.). Each *Agrobacterium* mixture was injected into 25 mulberry seedlings by pressure. A further 25 seedlings were injected with transformation buffer alone as a blank control. 

Thirty days after injection, the survival rate of seedlings in each group was greater than 70%, and the survival rates of seedlings in the three groups were 72.0%, 80.0%, and 76.0%, for the positive, mock and blank experimental groups, respectively. To assess the virus multiplication in the injected seedlings, PCR amplification of the tobacco brittle virus capsid protein *CP* gene sequence (Genbank No: Z36974.2) was performed (three replicates). From each treatment group, 16 seedlings with the same growth status were selected, divided into four groups (4 × 4) and cultivated in vermiculite nutrient soil watered with either 100 ppm, 200 ppm, 400 ppm or 800 ppm CuSO_4_. After 5 days, leaf samples were collected from each treatment (3 × 4 × 4) and immediately frozen in liquid nitrogen and stored at −80 °C for later RNA extraction, Cu concentration determinations and physiological analyses. 

#### 2.5.2. Quantification of Gene Expression by qRT-PCR

The qRT-PCR analysis was used to assess the expression level of *MaZIP4* in *MaZIP4*-VIGS plants treated with Cu in different concentrations based on the methods described previously. The relative expression differences of mRNAs were calculated using 2^−ΔΔCt^ method.

#### 2.5.3. Determination of Cu Concentration in Leaves

Leaves from WT, negative control and the *MaZIP4*-VIGS plants under different Cu stresses were washed with deionized water and dried, before digestion in a mixture of concentrated HNO_3_:H_2_O_2_ (3:2) in a microwave oven (Galanz, Shanghai, China). The concentrations of Cu were determined by inductively coupled plasma optical emission spectroscopy (ICP-OES, Avio 560 Max, Syngistix, PerkinElmer, Waltham, MA, USA).

#### 2.5.4. Determination of Physiological and Biochemical Indicators of Cu-Stress

Leaves of seedlings treated with Cu in different concentrations were collected to determine the content of soluble protein, free proline (PRO) and malondialdehyde (MDA), and to measure superoxide dismutase (SOD) and peroxidase (POD) activities as described in Liang et al. [33]. Each measurement was repeated three times. 

## 3. Results

### 3.1. Gene Expression Analysis and the Cloning of MaZIP4 

A total of 54,400,717 and 57,624,783 paired-end reads were obtained after sequencing all the libraries constructed from the Cu-treated and control plants on the Illumia NextSeq 500 platform, respectively (Table 1). After removing the low-quality reads, 53,546,796 and 56,701,537 clean reads were mapped to the reference mulberry genome, corresponding to more than 70% successfully mapped reads (Table 2). 

The comparison of the transcriptomes of Cu-treated and control plants indicated 5486 differentially expressed transcripts, 3078 of which were up-regulated and 2408 transcripts were down-regulated (Figure S2, Table S1. According to the transcriptome sequencing results, the expression of Zinc transporter 4 (*ZIP4*) gene was significantly upregulated after the Cu-treatment relative to the control group. 

The sequence of cloned *ZIP4-*like fragment, named *MaZIP4*, was 1405 bp in length with a full ORF (open reading frame) of 1254 bp (Figures S1 and S3), which was predicted to encode a 417-amino acid protein with a weight of 44.065 KD, the isoelectric point was 6.46. Protein sequence alignment analysis of Blast hits showed that the majority of high scoring hits were from members of the *ZIP* superfamily (Figure S4). Swiss-model software was used to predict the tertiary structure of the MaZIP4 protein (Figure S5), and the predicted results indicated that its spatial architecture was similar to that of ZIP4 proteins from other plants, suggesting that MaZIP4 may have similar functions. The amino-acid sequence alignment of *MaZIP4* protein show high homology with ZIP4 proteins of *Morus notabilis* (>80%), *Trema orientale*, *Ziziphus jujube* and *Cannabis sativa* (70%). This indicated that, although ZIP4 proteins show a high level of conservation among species, all these *ZIP4* proteins had a significantly long variant region (Figure S6).

**Figure 2:**
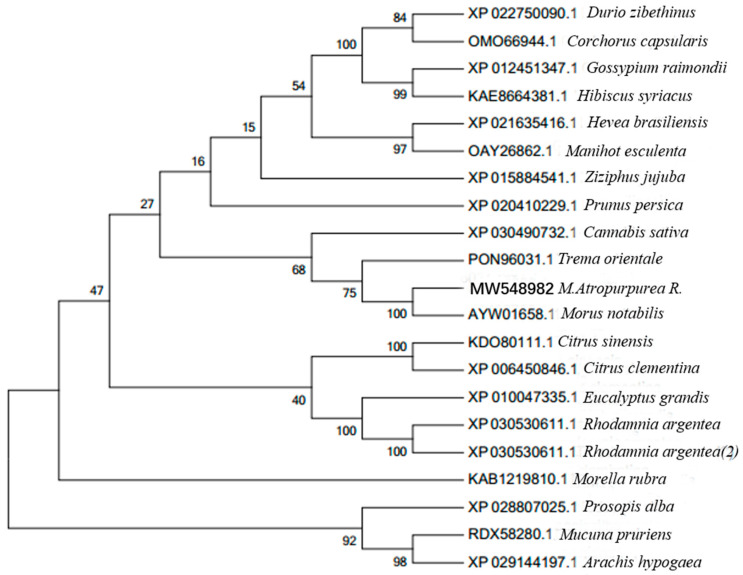
The phylogenetic tree based on the amino acid sequence of the *ZIP4* gene from mulberry (*M. atropurpurea* R.) and other homologous sequences from 19 different species. The protein accessions for the ZIP4 homologs and source species are given to the right of the figure. The evolutionary tree was established in the MEGA 6.0 program using the minimum-evolution test method. The numerals at the branch points indicate bootstrap percentages.

To further analyze the evolutionary relationship of ZIP4 members among species, a phylogenetic tree was constructed f their amino-acid sequences in mulberry and 19 other related species. The results revealed that *M. atropurpurea* R. showed a close evolutionary distance with *Morus notabilis* and *Trema orientale*, but was furthest from *Arachis hypogaea* and *Prosopis alba* (Figure 2). 

### 3.2. Expression of MaZIP4 under Cu Stress Treatments

**Figure 3:**
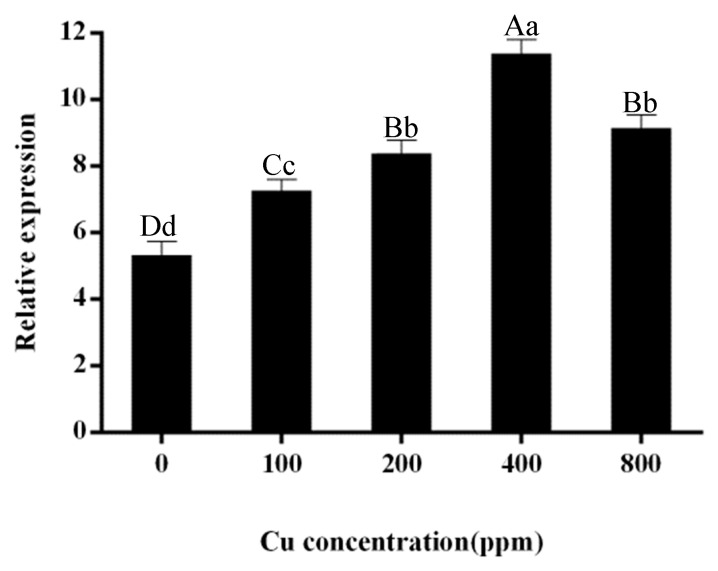
qRT-PCR measurements of the relative expression levels of *MaZIP4* gene in WT under different Cu concentrations. The error bars represent the mean ± SD of three replicates. Bars with different letters indicate a significant difference between expression levels at different concentrations (*p* ≤ 0.05) on the basis of Duncan’s multiple range test.

To further investigate the role of *MaZIP4* in Cu stress, the level of *MaZIP4* transcription under different concentrations of Cu stress was measured by qRT-PCR. The relative expression level of *MaZIP4* increased to a maximum at 400 ppm (11.35) and then decreased at 800 ppm (9.03), although maintaining a higher level than that of the control (0 ppm). This indicates that *MaZIP4* may have the ability to transport Cu within a certain range of Cu stress (Figure 3).

### 3.3. MaZIP4 Expression under Cu Stress after MaZIP4 Silencing

**Figure 4:**
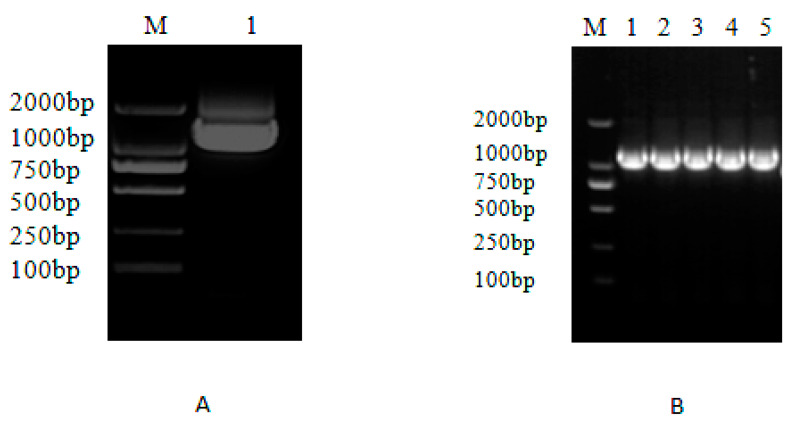
Construction and PCR detection of *MaZIP4* in the recombinant vector pTRV2-*MaZIP4* A: PCR amplification of *MaZIP4*. M: DL2000 bp marker, 1: *MaZIP4* B: PCR amplification of *MaZIP4* from the pTRV2-*MaZIP4* vector, M: molecular weight markers, 1–5: PCR of *MaZIP4*.

**Figure 5:**
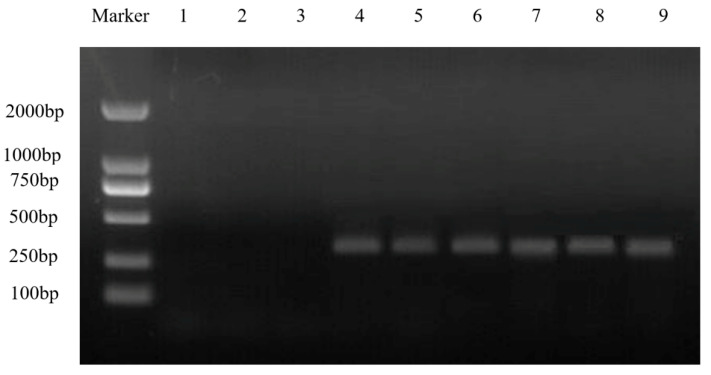
Infected plant virus expression results. Tobacco brittle virus capsid protein CP gene was not expressed in WT lines 1–3, and can be expressed normally in the negative control lines 4–6 and in *MaZIP4*-VIGS seedling lines 7–9. M: molecular weight markers.

To further verify the effect of *MaZIP4*, the construct pTRV2-*MaZIP4* was created for subsequent VIGS (Figure 4). To assess the proliferation of the virus, PCR amplification of the *CP* coat capsid gene was performed on randomly selected young leaf samples from the experimental group (pTRV2-*MaZIP4*), the negative control group (pTRV2), and the blank control group (WT; injected with transformation buffer alone). The results (Figure 5) indicated successful viral replication in the experimental and negative control groups, but not in the uninfected seedlings. 

**Figure 6:**
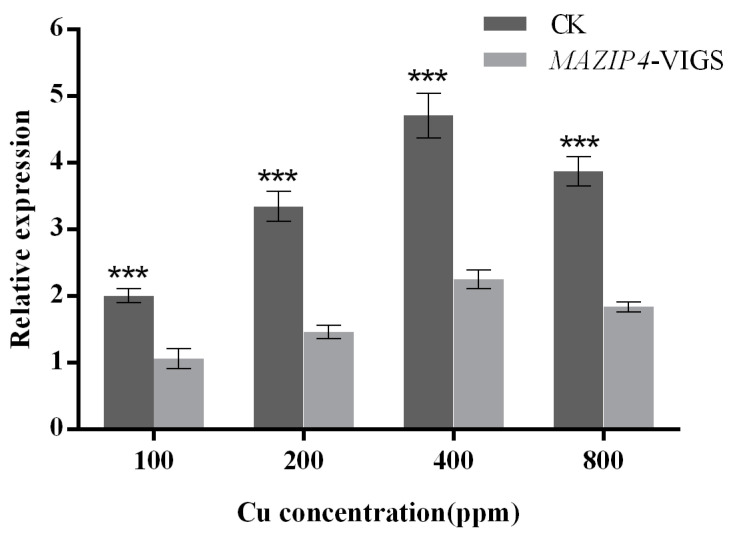
The expression level of *MaZIP4* gene under Cu stress after *MaZIP4* knock-down by VIGS. The error bars represent the mean ± standard deviation (SD) of the three biological replicates. Control means the negative control using the unaltered vector, pTRV2. *MaZIP4*-VIGS refers to the knock-down of *MaZIP4* gene in the experimental group. Asterisks indicate significant differences in expression levels between the control and *MaZIP4-*VIGS (*** *p* < 0.001), based on Duncan’s multiple range test.

qRT-PCR analysis was used to assess the expression levels of *MaZIP4* in *MaZIP4*-VIGS plants treated with Cu in different concentrations (Figure 4 and Figure 6). Consistent with that observed in WT plants, the expression of *MaZIP4* in *MaZIP4*-VIGS plants increased up to 400 ppm Cu then decreased at 800 ppm Cu. However, the expression levels of *MaZIP4* were consistently reduced to about 50% of that in WT plants, indicating that *MaZIP4* was successfully knocked down in *MaZIP4*-VIGS plants. 

### 3.4. MaZIP4 Silencing Increased Cu Accumulation in Cu Treated Mulberry Leaves 

**Figure 7:**
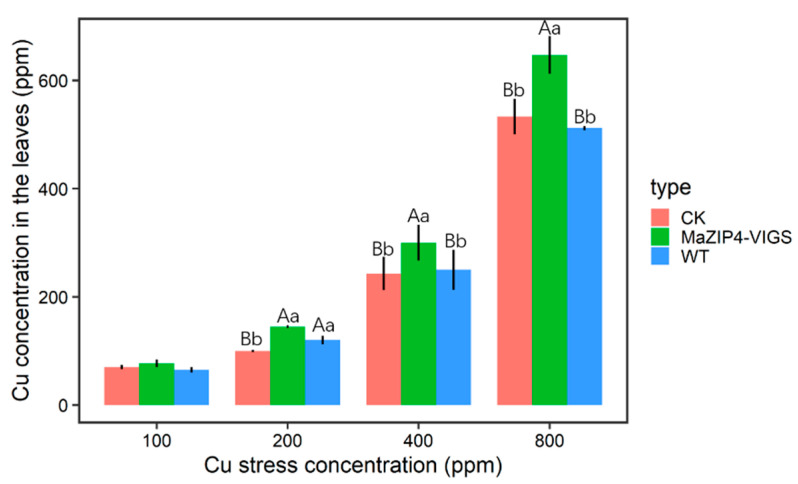
Mean values of Cu concentration in leaves of CK, MaZIP4-VIGS and WT plants under different Cu stress concentrations. Error bars represent the mean ± SD of the three biological replicates. Different lowercase letters above bar represent significant differences (*p <* 0.05, one-way ANOVA). Different capital letters above bars indicate significant differences (*p* < 0.001, one-way ANOVA). The same letter or no letter above bars indicates no significant difference.

**Figure 8:**
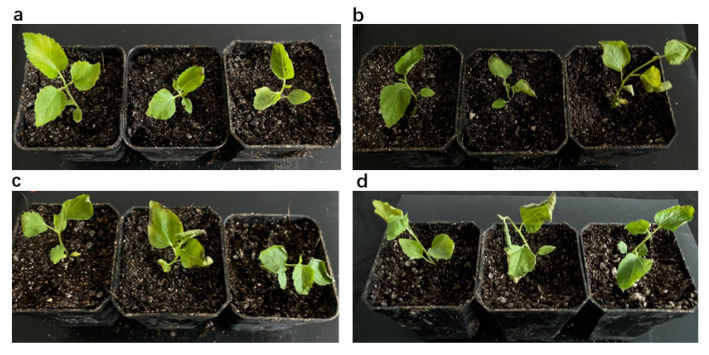
The changes in phenotypes of the blank control (WT), the negative control [2] and *MaZIP4-*VIGS plants under different copper stress concentrations. (**a**) coercive concentration of 100 ppm, (**b**) coercive concentration of 200 ppm, (**c**) coercive concentration of 400 ppm, (**d**) coercive concentration of 800 ppm. And from left to right in one photo are CK, *MaZIP4-VIGS* plants and WT.

The Cu concentration in leaves was higher in *MaZIP4*-VIGS plants than the blank and negative controls, and increased with the intensity of the Cu stress applied over 0–400 ppm. It is obvious that MaZIP4 gene is likely to be involved in the maintaining the homeostasis of Cu^2+^ in leaves (Figure 7). In addition, with the increase in Cu concentration, mulberry leaves showed higher degrees of yellowing. At 800 ppm Cu, the WT, negative control and *MaZIP4*-VIGS plants all showed obvious leaf yellowing and shrinkage (Figure 8) However, it is clear that relative to the negative and blank controls, the reduced expression of *MaZIP4* in *MaZIP4*-VIGS lines resulted in a significantly greater Cu accumulation in leaves in plants exposed to 400 and 800 ppm Cu, thus supporting a role for *MaZIP4* in Cu homeostasis.

### 3.5. MaZIP4 Silencing Induced Physiological and Biochemical Changes in Mulberry Associated with Cu Stress

**Figure 9:**
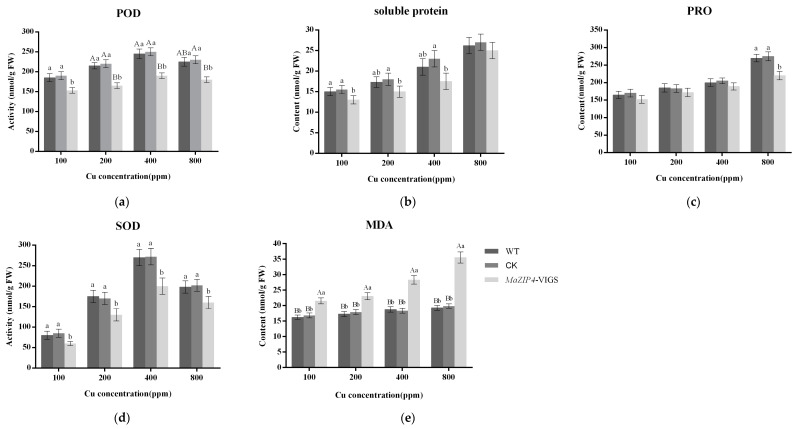
Analysis of contents of soluble protein, PRO, MDA and activities of SOD, POD in mulberry leaves of *MaZIP4*-VIGS lines (light grey bars), negative control (gray bars) and WT (dark grey bars) exposed to various concentrations of Cu. Bars represent mean ± SD (*n* = 3). Different lowercase letters above bar represent significant differences (*p* < 0.05, one-way ANOVA); Different capital letters mean significant differences (*p* < 0.001, one-way ANOVA); The same letter and no letter above bars mean no significant difference. (**a**) POD. (**b**) soluble protein. (**c**) proline. (**d**) SOD. (**e**) MDA.

Several important physiological and biochemical indicators related to plant stress responses were compared between in the blank control, negative control and *MaZIP4*-VIGS mulberry plants. MDA concentration is commonly used to measure the degree of lipid peroxidation caused by the stress-induced accumulation of reactive oxygen species (ROS) (Figure 9e). The MDA content of *MaZIP4*-VIGS plants, negative control and WT lines gradually increased in all treatment groups with increasing concentrations of Cu stress treatment. Relative to the negative control and the WT, the MDA content in *MaZIP4*-VIGS plants increased rapidly and reached a maximum at 800 ppm Cu (Figure 9e). This indicates that WT levels *MaZIP4* can more effectively inhibit the increase in MDA content in mulberry under Cu stress. The activity of POD and SOD in plants is often considered to be related to the plant stress response. Under abiotic stress, antioxidant enzymes in plants such as POD and SOD promote stress tolerance by reducing stress-induced accumulation of ROS. The POD and SOD activities in WT and the negative control groups were enhanced with increasing concentration of Cu up to 400 ppm and showed a reduced increase at 800 ppm. However, the activity of both enzymes in the *MaZIP4*-VIGS transgenic plants was lower than that of WT and negative control group under the 4 different Cu treatment concentrations tested, and reached their maximum at 400 ppm Cu (Figure 9a,d). These results suggest that the mulberry response to Cu stress involves an increase in POD and SOD activities to help reduce ROS levels. The reduced mobilization of this response in *MaZIP4*-VIGS lines also suggests that *MaZIP4* expression is required for the positive regulation of this response. PRO is a common osmolyte which widely exists in tissues of plants and accumulates in response to abiotic stress (heat, cold, salinity, drought) and is often positively correlated with plant resistance. In WT, negative control lines and *MaZIP4*-VIGS plants, the PRO content increased with increasing of Cu concentration and reached a maximum at 800 ppm. Relative to the WT and negative control, the PRO content in *MaZIP4*-VIGS plants was the lowest, and showed no correlation with the Cu concentration (Figure 9c). Similar to that observed for POD and SOD activities, these results also indicate that PRO accumulation reflects Cu-stress and that PRO accumulation is positively regulated under such conditions by *MaZIP4*. The content of soluble protein accumulation in leaves in WT, negative control lines and *MaZIP4*-VIGS plants all increased gradually with increasing Cu concentration over the range 0–800 ppm. The soluble protein content of *MaZIP4*-VIGS plants also increased with increasing copper concentration, but in lower levels than that observed in WT and negative control lines at all Cu treatments (Figure 9b). This suggested that soluble proteins may be involved in the copper stress response in mulberry and that *MaZIP4* may be required for this response.

## 4. Discussion

The heavy metal Cu is one of essential micronutrients for plant growth and development, but can be toxic in excess. To prevent excess metal toxicity, plants evolved various mechanisms for regulating metal uptake and transport. ZIP family proteins belong to a family of metal transporters involved in the uptake and transportation of Cu, Zn, Ca, Fe and Mn and play vital roles in the maintenance of metal homeostasis in plant tissues [23,34,35]. In our study, a *ZIP4* gene homolog (*MaZIP4*) was identified and cloned for the first time in *M*. *atropurpurea* R. We further characterized the *MaZIP4* gene to explore its potential function in the mulberry response to Cu stress.

A comparative transcriptome analysis indicated that 3078 transcripts were significantly up-regulated in mulberry in response to treatment with 200 ppm CuSO_4_, including *MaZIP4*, suggesting The *MaZIP4* gene is 1405 bp in length with a full ORF of 1245 bp and encodes a 417 amino acid protein. Homology and evolutionary analyses indicated that the MaZIP4 showed more than 67% sequence identity with ZIPs of 19 other plant species. The high degree of sequence structural similarities between *MaZIP4* and other ZIP proteins suggests they share similar biological functions. The expression of some zinc-regulated ZIP transporters is also up-regulated under Cu stress, and ZIPs have long been considered responsible for the uptake and allocation of Cu in *Arabidopsis* [14,35]. Therefore, we questioned whether *MaZIP4* might be involved in Cu^2+^ uptake and transport in Mulberry. In support of this, qRT-PCR analyses indicated that with the increase of Cu stress up to 400 ppm, the expression level of *MaZIP4* was significantly enhanced. However, with further increases in Cu (400–800 pm), the expression of *MaZIP4* was decreased, suggesting that the regulatory effect of the gene on Cu stress was limited to a certain range. To further test the function of *MaZIP4*, pTRV2*-MaZIP4* VIGS lines of mulberry (*MaZIP4-*VIGS). qRT-PCR analysis showed the expression level of *MaZIP4* in the transgenic lines was reduced to 50% of the negative control and WT lines under different levels of Cu stress. We next wished to compare physiological and biochemical indicators of the stress response in *MaZIP4*-VIGS, WT and negative control lines subjected to different degrees of Cu stress. When subjected to abiotic stress, plants produce reactive oxygen species (ROS) [36], leading to the peroxidation of membrane lipids which is often assayed indirectly by measuring MDA. MDA is a widely used marker of environmental stress in plants [37]. The content of MDA and the activity of antioxidant enzymes is expected to increase initially and then decrease due to excessive Cu [38,39,40], which is consistent with our findings. Zhou et al. (2015) studied the changes of physiological and biochemical reactions of forest trees under different levels of Pb stress, and showed that the activities of superoxide dismutase (SOD), peroxidase (POD) and MDA contents in trees were significantly increased under Pb stress [41]. SODs catalyze the dismutation of superoxide into molecular oxygen and hydrogen peroxide which is subsequently catalyzed to H_2_O by peroxidase enzymes (POD), and are often used as an indicator of abiotic stress tolerance [42]. The upregulation of SODs and POD has been reported in many plant species including mulberry under abiotic stresses, including drought, salt, and heavy metals [43,44,45,46,47,48]. The content of PRO is often considered a biomarker of stress because it accumulates under adverse environmental conditions. The accumulation of soluble protein is generally considered a signal of metabolic disorder but not simply a response to stress. Although the change of physiological and biochemical state is not the only way for plants to cope with the environment stress, plants with higher SOD, soluble protein, PRO, POD and lower MDA usually display higher stress resistance [49,50]. Our results showed that the activities of SOD, POD and MDA, soluble protein, PRO contents in mulberry were significantly increased under Cu stress, indicating that Cu stress may stimulate the mobilization of the stress response in mulberry to reduce heavy metal damages. However, while the response to Cu stress in transient transgenic lines for the reduced expression of *MaZIP4* showed higher values for MDA content, these lines also showed lower activities of POD and SOD activities, as well as PRO and soluble protein contents relative to WT and negative control lines. This indicates that the reduced expression of *MaZIP4* in response to Cu stress occurred with the repression of aspects of the plant response to stress. These results indicate that *MaZIP4* may directly contribute to mulberry resistance to Cu stress through its role as a Cu transporter, but that it may also indirectly contribute to the response to Cu stress via a regulatory role in the mobilization of the general stress response. However, further studies are required to elucidate the underlying mechanisms involved in the mitigating role of *MaZIP4* in mulberry under Cu stress. 

## 5. Conclusions

Transcriptome sequencing yielded a new *ZIP4* gene (*MaZIP4*), which was isolated and cloned from *M. atropurpurea* Roxb. This is also the first time a zinc transporter 4 member has been cloned from *M. atropurpurea* Roxb. Phylogenetic analysis indicated that MaZIP4 shows high homology with ZIP4 proteins of *Morus notabilis*, *Trema orientale*, *Ziziphus jujube* and *Cannabis sativa*. The expression of *MaZIP4* in mulberry was increased under a certain degree of Cu stress, which may indicate that *MaZIP4* gene may be involved with the transport of Cu ions in mulberry to maintain Cu homeostasis. After *MaZIP4* knock-down by VIGS, the expression of *MaZIP4* was reduced to about 50% of WT plants (Young et al., 2010). Physiological analyses of *MaZIP4*-VIGS land negative control lines revealed that the partial repression of *MaZIP4* resulted in an increased leaf content of MDA, whereas the contents of soluble protein and proline, as well as the activities of SOD and POD), were decreased. These results indicated that *MaZIP4* could enhance Cu tolerance through its function as a Cu transporter, but also as a positive regulator of the general stress response in mulberry. Our study provides preliminary evidence that *MaZIP4* may have a critical role in regulating Cu^2+^ homeostasis in mulberry and lays the foundation for future studies into the mechanism underlying the plant response to Cu stress.

## Figures and Tables

**Table 1:** Statistical table of sequencing data quality.

Sample Name	Raw Reads	Clean Reads	Clean Bases	Error Rate (%)	Q20 (%)	Q30 (%)	GC Content (%)
Control	57,624,783	56,701,537	8.51G	0.03	96.76	91.62	45.54
Cu stress	54,400,717	53,546,796	8.04G	0.03	96.84	91.78	45.96

**Table 2:** Statistical table of the comparison between reads and reference genome.

Sample Name	Total Reads	Total Mapped	Multiple Mapped	Uniquely Mapped	Reads Map to ‘+’	Reads Map to ‘−’
Control	56,701,537	40,105,596 (70.73%)	1,528,234 (2.69%)	38,577,363 (68.04%)	19,238,895 (33.93%)	19,338,468 (34.11%)
Cu stress	53,546,796	39,001,494 (72.84%)	1,439,133 (2.69%)	37,562,362 (70.15%)	18,764,150 (35.05%)	18,798,212 (35.11%)

## Data Availability

The data presented in this study are available in supplementary material here.

## Supplementary Materials

The following supporting information can be downloaded at: https://www.mdpi.com/article/10.3390/life12091311/s1, Figure S1: MaZIP4 gene amplification of mulberry: PCR in vitro amplification was performed using the designed primers MaZIP4-F and MaZIP4-R, and gene product band of about 1200 bp in length was obtained. M: DL2000 DNA molecular marker 1: Product of RT-PCR; Figure S2. Volcano map of differential genes; Figure S3. *MaZIP4* gene cDNA sequence and translated amino acid sequence; Figure S4. Prediction of amino acid sequence encoded by *MaZIP4* gene; Figure S5. *MaZIP4* gene protein tertiary structure; Figure S6. *MaZIP4* encode multiple sequence alignments of amino acids; Table S1: Screening of differential genes and their corresponding primers.

## Abbreviations

TM3: transmembrane domains III; TM4: transmembrane domains IV; VIGS: virus-induced gene silencing; FPKM: The fragments per kilobase of transcript per million mapped reads; ORF: open reading frame; MDA: malondialdehyde; PRO: proline; SOD: superoxide dismutase; POD: peroxidase; PI: isoelectric point; AS: acetosyringone; CK: the negative control inoculated with empty vector pTRV2; WT: the blank control.
