# Supplementary material for: Functional Characterization of MaZIP4, a Gene Regulating Copper Stress Tolerance in Mulberry (Morus atropurpurea R.)
Source: Life (Basel). 2022 Aug 26;12(9):1311. doi: 10.3390/life12091311 (PMC9505184; doi:10.3390/life12091311)
Supplement: Supplementary file 1 [file life-12-01311-s001.zip › life-1875631-Supplementary.pdf]

Supplement Materials

Table S1 Screening of differential genes and their corresponding primers

Gene_id	Regulated	Name	Primer	Primer sequence(5'-3')
21390692	up	F-box protein PP2-B15	Forward	CGATCGCGCATATGGATTGG
			Reverse	ACCTTGACCTCAAAGCCTCG
21387802	up	Purple acid phosphatase 17	Forward	ATTGGAGGGGTATTTCGCCG
			Reverse	TCCTTCGTGTCACCGTGATG
21403433	up	ATP-dependent DNA helicase MER3 homolog	Forward	TCGAGGTTCCTGTTCAAGGC
21401914	up	Cytochrome P450 71A1	Reverse	AGGCCATTGCTGACTGAGAC
			Forward	TGTGGCCGTACATCTCAATCC
21402441	up	Zinc transporter 4	Reverse	CTGTTGATGTGCCAGCCTCT
			Forward	GTGAGGGAGCATTCACACGG
21387250	up	Pathogenesis-related protein 1	Reverse	TCTTTCTGCCCACTGAGAAACG
			Forward	CATAGGCCTAGCCATGCTCC
21397686	up	Putative disease resistance protein	Reverse	TTGTCGTCCCACGTCATCTG
			Forward	AAGCTTCGGTTAGTGCTCCG
21405925	up	Leucoanthocyanidin dioxygenase	Reverse	TTTGCGCTCTTGTGCTTCAG
			Forward	CCATGGTATCCCTGACGAGC
21399859	up	Bark storage protein A	Reverse	TTGTTTGCGAGCTTGCTTCC
			Forward	GTCTCCAAGGGAAGCCCAAG
21395828	up	Receptor-like protein 12	Reverse	ACAGCCGAACTCTCCATGTC
			Forward	CCTTTCTCGACCTAGGGCAC
21396136	down	Probable protein phosphatase 2C 75	Reverse	CGACGAGATTGTTGTGAGCG
			Forward	AGTACTGCCGGAGAATGCTG
21405820	down	(R)-mandelonitrile lyase-like	Reverse	CTTGCCGCTTCCAACTGTTC
			Forward	TGCTACCGGGATTGGAAGTG
21394160	down	Probable protein phosphatase 2C 6	Reverse	CGGCAACGAAAGGAGACTCA
			Forward	GGTCATGACGGAGACAGTCG
21385141	down	Protein CLT3	Reverse	TCCCTCGACAAAGAACTGCC
			Forward	TGAAGTGCTGGAACCCAGTG
21384129	down	Wall-associated receptor kinase 2	Reverse	CTGTATCAGCGGCTTGGAAC
			Forward	GAAACGCTTACCTCCCTGCT
β-actin			Forward	AGCAACTGGGATGACATGGAGA
			Reverse	CGACCACTGGCGTAAAGGGA

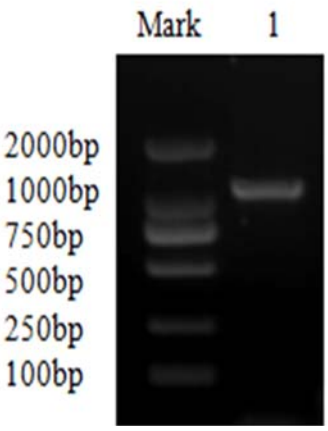

Figure S1. MaZIP4 gene amplification of mulberry: PCR in vitro amplification was performed using the designed primers MaZIP4-F and MaZIP4-R, and gene product band of about 1200 bp in length was obtained. M: DL2000 DNA molecular marker 1: Product of RT-PCR.

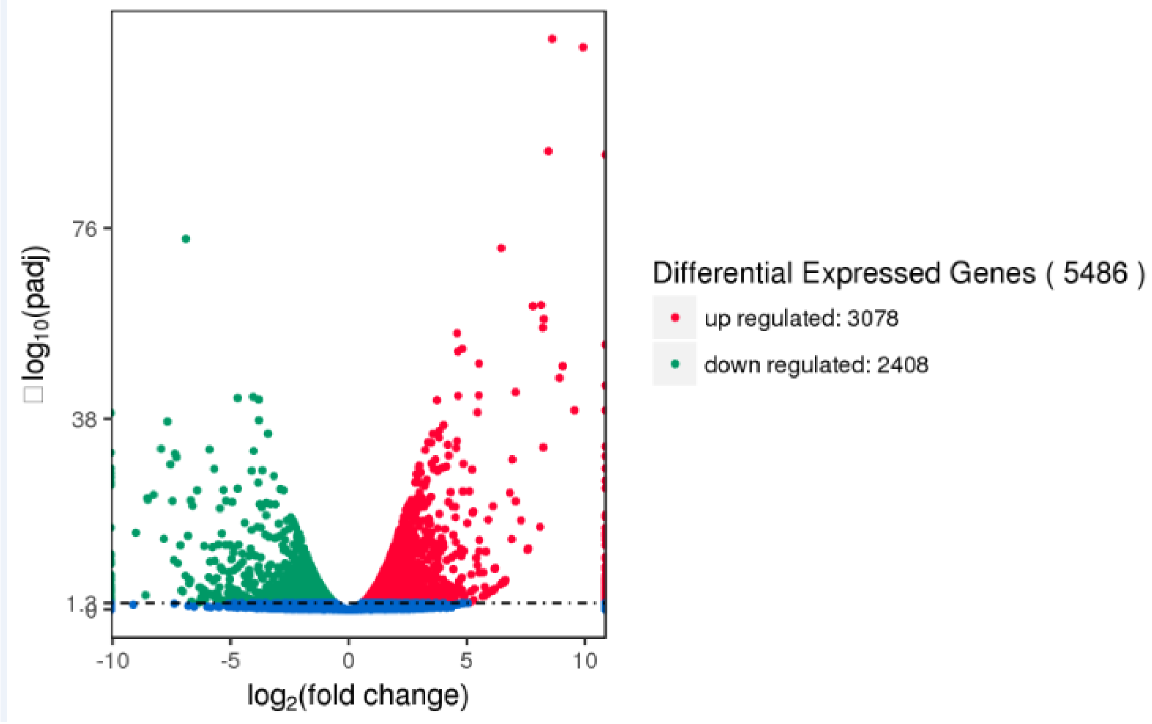

Figure S2. Volcano map of differential genes. The overall distribution of DEGs is visualized with a volcano plot, and genes with significant differential expression are indicated by red dots (up-regulated) and green dots (down-regulated), while genes with insignificant differential expression are indicated by blue dots.

```
     ATGGGCAGCAGCCATCATCATCATCATCACAGCAGCGGCCTGGTGCCGCGCGGCAGCCAT
1     M  G  S  S  H  H  H  H  H  H  S  S  G  L  V  P  R  G  S  H
61   ATGGCTAGCATGACTGGTGGACAGCAAATGGGTCGCGGATCCATGGCGAATACAAGTTGC
21    M  A  S  M  T  G  G  Q  Q  M  G  R  G  S  M  A  N  T  S  C
121  CAGAGCGCGGAGCTAGAGGTATGCCGGGATGAATCGGTGGCGCGCGTCCTCAAATTCGTC
41    Q  S  A  E  L  E  V  C  R  D  E  S  V  A  R  V  L  K  F  V
181  GCCATCGCCTCGATCCTCCTCTCCGGAGTGGCCGGAGTCGCCATTCCCCTCATTGGAAAG
61    A  I  A  S  I  L  L  S  G  V  A  G  V  A  I  P  L  I  G  K
241  CACCGCCGGTTTCTCCGCACCGATGGCTGCCTCTTCGTCGCCGCCAAGGCCTTCGCCGCC
81    H  R  R  F  L  R  T  D  G  C  L  F  V  A  A  K  A  F  A  A
301  GGCGTCATCCTGGCCACCGGGTTCGTCCACATGCTCTCAGGTGGGTCGGACGCGCTATCC
101   G  V  I  L  A  T  G  F  V  H  M  L  S  G  G  S  D  A  L  S
361  AATCCTTGCCTGCCCGAGTACCCGTGGGCTAAGTTTCCTTTCTGTGGGTTCTTCGCGATG
121   N  P  C  L  P  E  Y  P  W  A  K  F  P  F  C  G  F  F  A  M
421  ATGGCCTCGCTGTTGACCTTACTCGTTGACTTTATGGGCACTCAGTATTACGAGAAGAAG
141   M  A  S  L  L  T  L  L  V  D  F  M  G  T  Q  Y  Y  E  K  K
481  CAAGGGCTGGCCCGGTCCAGCAGCGAGGAGCAAGCTCGGGTCGGATCGGAGGATTCGGGT
161   Q  G  L  A  R  S  S  S  E  E  Q  A  R  V  G  S  E  D  S  G
541  GGTGAAACCGGAATTGTTGCGGTTCAGGCCAATGATTGGAATGGGAAGGTGTTCGGAGAA
181   G  E  T  G  I  V  A  V  Q  A  N  D  W  N  G  K  V  F  G  E
601  GAAGAGGGTGGTGGTATGCACATTGTGGGAATGCATGCGCACGCTGCTCACCATAGACAC
201   E  E  G  G  G  M  H  I  V  G  M  H  A  H  A  A  H  H  R  H
661  AGTCATGCTCAAGAGCACGGTGCATGTGATGGGCACGTGAAGGAGCATTCACACGGGCAC
221   S  H  A  Q  E  H  G  A  C  D  G  H  V  K  E  H  S  H  G  H
721  GGGCATGACCACGGGCACTCGCATGGGTTGAGAGATGGGGACGAGGAAGGTGGTGTTAGG
241   G  H  D  H  G  H  S  H  G  L  R  D  G  D  E  E  G  G  V  R
781  CACGTCGTCGTTTCTCAGGTTTTGGAACTTGGGATTGTATCACATTCAGTTATAATCGGG
261   H  V  V  V  S  Q  V  L  E  L  G  I  V  S  H  S  V  I  I  G
841  CTATCTCTCGGAGTCTCGCAGAGTCCATGTACAATAAGACCCTTAATTGCTGCATTATCC
281   L  S  L  G  V  S  Q  S  P  C  T  I  R  P  L  I  A  A  L  S
901  TTCCACCAGTTCTTCGAAGGATTTGCACTCGGAGGCTGCATCGCCCAAGCCCAATTCAAG
301   F  H  Q  F  F  E  G  F  A  L  G  G  C  I  A  Q  A  Q  F  K
961  ACCCTTTCGGCCACAATAATGGCATGCTTTTTCGCGATAACTACTCCCGCGGGGATTGGC
321   T  L  S  A  T  I  M  A  C  F  F  A  I  T  T  P  A  G  I  G
1021 ATTGGGACTGCCATTGCTTCTTTCTACAACCCGTATAGCACTGGAGCATTGGTTACTGAA
341   I  G  T  A  I  A  S  F  Y  N  P  Y  S  T  G  A  L  V  T  E
1081 GGCATTTTGGATTCTTTGTCAGCTGGGATTCTAGTCTACATGGCTTTGGTGGACCTGATT
361   G  I  L  D  S  L  S  A  G  I  L  V  Y  M  A  L  V  D  L  I
1141 GCAGCTGATTTTTTGAGCAAGAGGATGAGCTGCAATTTTAGGCTGCAAGTGGCATCCTAT
381   A  A  D  F  L  S  K  R  M  S  C  N  F  R  L  Q  V  A  S  Y
1201 TTAATGCTTTTCCTAGGCGCTGGGTTGATGTCTTCATTAGCTATTTGGGCTTGA
401   L  M  L  F  L  G  A  G  L  M  S  S  L  A  I  W  A  *
```

ATG: the start codon; TGA: the stop codon

Figure S3. *MaZIP4* gene cDNA sequence and translated amino acid sequence. Sequence analysis of the cloned *MaZIP4* gene using DNAMAN software showed that the sequence length of the *MaZIP4* gene was 1405 bp, containing a complete ORF of 1254 bp, which can encode a protein with 417 amino acids.

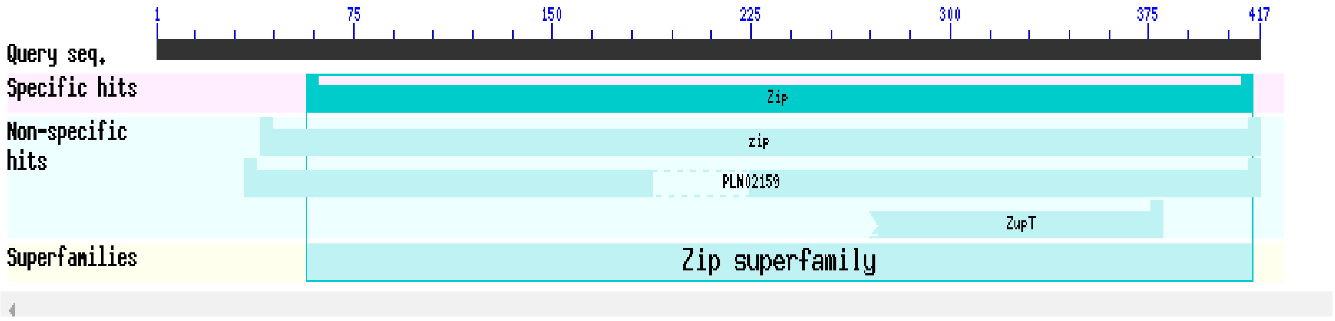

Figure S4. Prediction of amino acid sequence encoded by *MaZIP4* gene. NCBI online Blast results indicate that the amino acid family encoded by the *MaZIP4* gene belongs to the *ZIP4* superfamily.

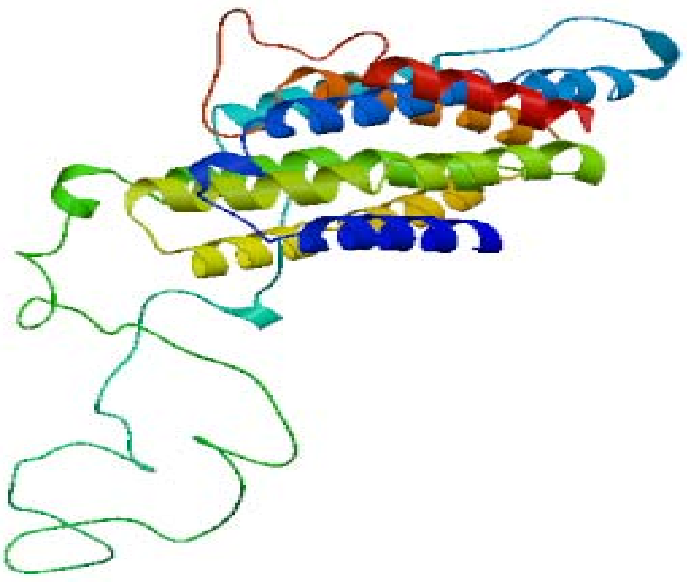

Figure S5. *MaZIP4* gene protein tertiary structure. Since the tertiary structure of the *MaZIP4*-encoded protein is similar to that of *ZIP* family proteins, it is speculated that: the *MaZIP4*-encoded protein may have a similar function to that of *ZIP* family proteins.

```
M.atropurpurea  MGSSHHHHHHSSGLVPRGSHMASMTGGQQMGRGSMANTSC
M.notabilis     ..................................MANTSC
Trema           MLLLEDIWQFLCLEGFVDKIRLYPGTLLQTVSDSMTNASC
Hevea           MLFFEDLWELIGLNHFTLKTRALSESLFPGISESMSNSSC
Ziziphus        MLFIEDLWPLLGLRGFVGKAQGFSDSILQTISLSMTNASC
Durio           MLFIEDIWPLFSIGCFGSKTKLFSESLLQSITDSMSNTSC
Cannabis        MLFLEELWQFLSLGGFVDKTRLSAGFFLQTVSESMANSTC
Corchorus       ..................................MTNSSC
Consensus                                         m n  c

M.atropurpurea  HRRFLRTDGCLFVAAKAFAAGVILATGFVHMLSGGSDALS
M.notabilis     HRRFLRTDGCLFVAAKAFAAGVILATGFVHMLSGGSDALS
Trema           RRRFLRTDGSLFVAAKAFAAGVILATGFVHMLSGGSEALS
Hevea           QRRFLRTDGSLFVAAKAFAAGVILATGFVHMLSGGSNALR
Ziziphus        QRSFLRTDGSLFVAAKAFAAGVILATGFVHMLSGGSDALS
Durio           QRKFLRTDGSLFVAAKAFAAGVILATGFVHMLSAGTDALT
Cannabis        HRRLLRTDGSLFVAAKAFAAGVILSTGFVHMLSGGSEALS
Corchorus       HRKFLRTDGSLFVAAKAFAAGVILATGFVHMLTGGTDALT
Consensus        r  lrtdg lfvaakafaagvil tgfvhml  g  al

M.atropurpurea  NPCLPEYPWAKFPFCGFFAMMASLLTLLVDFMGTQYYEKK
M.notabilis     NPCLPEYPWAKFPFCGFFAMMASLLTLLVDFMGTQYYEKK
Trema           NPCLPEYPWAKFPFCGFFAMMASLLTLLVDFVGTQYYERK
Hevea           NPCLPEYPWSKFPFPGFIAMMASLLTLLVDFVGTQYYERK
Ziziphus        NPCLPEYPWSKFPFCGFFAMLASLVTLLVDFVGTQYYERK
Durio           DPCLPKYPWSKFPFSGFFAMIASLITLLVDFVGTQYYEKK
Cannabis        NPCLPKFPWSKFPFCGFFAMVAALLTLLVDFVGTQYYERK
Corchorus       NPCLPKYPWSKFPFSGFFAMIAALLTLLVDFVGTQYYERK
Consensus        pclp  pw kfpf gf am a l tllvdf gtqyye k

M.atropurpurea  QG.LARSSSEEQARVGSEDSGGETG.IVA.VQANDWNGKV
M.notabilis     QG.LARPSSEEQARVGSDDSGGETG.IVA.LQANDWNGKV
Trema           QG.LARPNSEDQGRVGSEDSGMESG.IVPVVQAKEWNGKV
Hevea           QG.LNR.ASEEQARVGSVDVGPDSD.IVPFAGEKEPNGKV
Ziziphus        QG.LAKP.SEERVRVGFDDSGLEST.IVP.AQAKEWNGKV
Durio           QG.LARGKTEEQGRVESLEADSESA.IVPVVEGKDWNGKV
Cannabis        QGKKTRPNGEEQSRVGSGED..ESG.IVR.VGSKEWNGKV
Corchorus       QG.LARGSTEEQARVLSIEADSESAGIMPVLEAKDRNGKV
Consensus       gg       e   rv           i         ngkv

M.atropurpurea  FGEEEGGGMHIVGMHAHAAHHRHSHAQEHGACDGHVKE..
M.notabilis     FGEEEGGGMHIVGMHAHAAHHRHSHAQEHGACDGHVRE..
Trema           FGEEEGGGMHIVGMHAHAAHHRHNHPQEQGACDGHVRE..
Hevea           FGEEEGGGMHIVGMHAHAAHHRDSHPHGQDACDGHVK...
Ziziphus        FGEEESGGMHIVGMHAHAAHHRHNHPHEQGACDGHVREQS
Durio           FGEEEGGGMHIVGMHAHAAHHRHSHPHGQDGCDGLLRSRG
Cannabis        FGEEEGGGMHIVGMHAHAAHHRHSHGQEQGACDGHVTE..
Corchorus       FGEEEGGGMHIVGMHAHAAHHRHNHPHGQDGCDGLLRSRG
Consensus       fgeee ggmhivgmhahaahhr  h      cdg

M.atropurpurea  ....HSHGHG.....HDHGHSHGLRDGDEEGGVRHVVVSQ
M.notabilis     ....HSHGHG.....HDHGHSHGLGDGNEEGGVRHVVVSQ
Trema           ....NSX..........XGHSHGFGDGDEEGGVRHVVVSQ
Hevea           .....DHGHG.........HNHRFSEGDEESGARHVAVSQ
Ziziphus        HGHEHGHGHG.....HGHGHSHGLGE.DEDSGVRHVVVSQ
Durio           ....HEHDH.........GHEHGFGGGDADNGVRHVVVSQ
Cannabis        ....VGHGHG.....HGHGHGHGFGDGDEEGGVRHVVVSQ
Corchorus       ....HEHDHGQHGHSHGHGHDHGFGGSDDDSGVRHVVVSQ
Consensus                          h h         g rhv vsq
```

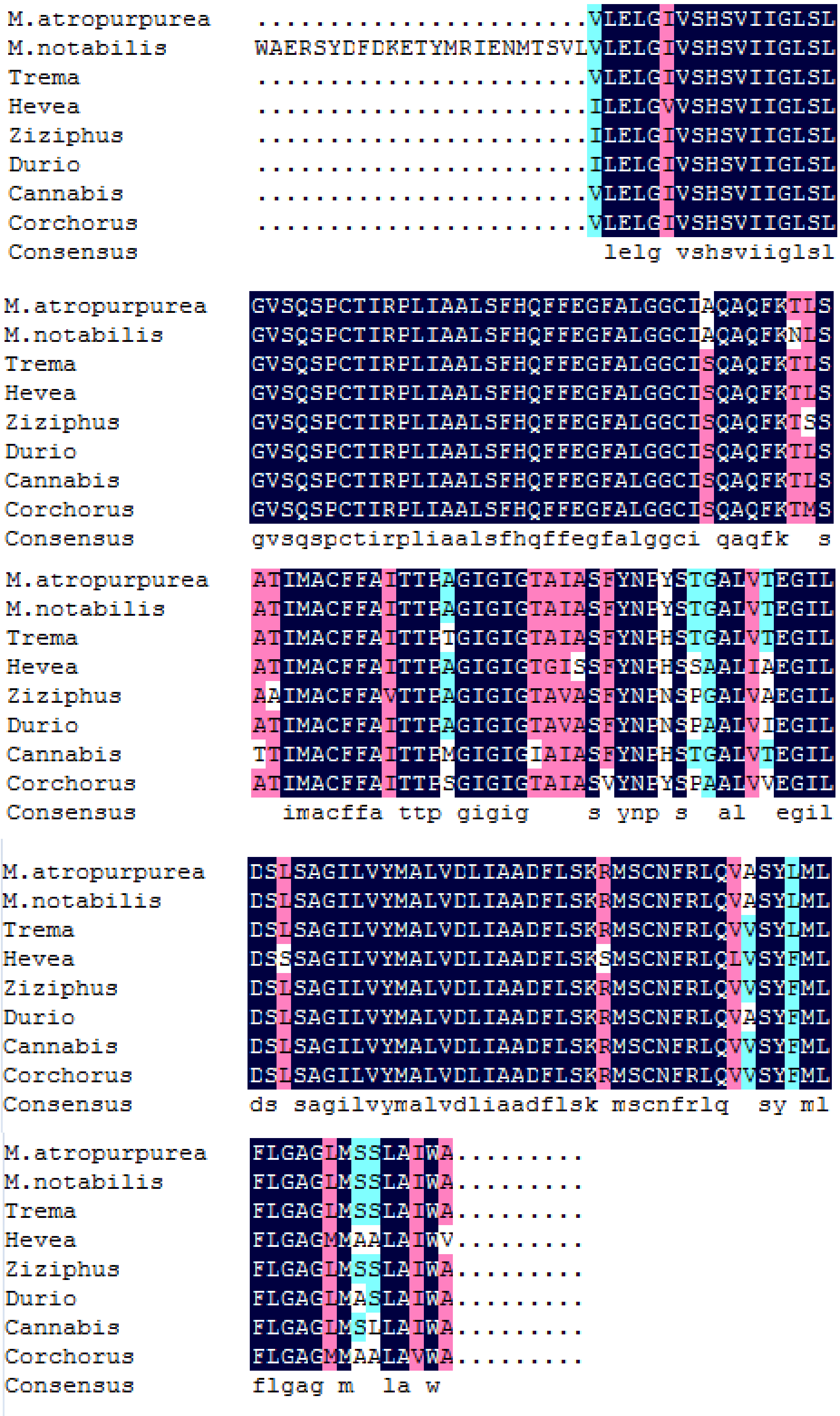

Figure S6. *MaZIP4* encode multiple sequence alignments of amino acids. Sequence alignment of the mazip4-encoded protein sequence in *M. atropurpurea R.* was performed using the multiple sequence alignment function in the online site NCBI.
